# Phototaxis and Motility of *Euglena gracilis* in Physiological Saline via Stepwise Acclimation for Biohybrid Microrobotics

**DOI:** 10.3390/mi17070815

**Published:** 2026-07-06

**Authors:** Kaiya Endo, Soshi Morimoto, Hayato Obayashi, Takayuki Shibata, Shunya Okamoto, Tuhin Subhra Santra, Moeto Nagai

**Affiliations:** 1 Department of Mechanical Engineering, Toyohashi University of Technology, Toyohashi-shi 441-8580, Japan; endo.kaiya.tb@tut.jp (K.E.); morimoto.soshi.qa@tut.jp (S.M.); obayashi.hayato.mi@tut.jp (H.O.); shibata@me.tut.ac.jp (T.S.); okamoto@me.tut.ac.jp (S.O.); 2 Department of Engineering Design, Indian Institute of Technology Madras, Chennai 600036, India; tuhin@smail.iitm.ac.in; 3 The Institute for Research on Next-generation Semiconductor and Sensing Science (IRES^2^), Toyohashi University of Technology, Toyohashi-shi 441-8580, Japan

**Keywords:** *Euglena gracilis*, microrobot, phototaxis, acclimation

## Abstract

Microrobots navigating the human body require biocompatible actuators capable of functioning in physiological fluids. The microalga *Euglena gracilis* offers precise phototactic control; however, its operational stability in simulated physiological environments remains unproven. Here, we report that a stepwise acclimation process preserves the robotic functionality of *E. gracilis* in 100% phosphate-buffered saline (PBS), 100% fetal bovine serum (FBS), and a NaCl solution at a concentration equivalent to PBS (137 mM). We compared direct transfer against a graduated adaptation protocol, evaluating morphology, swimming speed, motility rate, and phototaxis. Direct transfer to each medium caused near-total immobilization, whereas stepwise acclimation retained motility. Acclimated cells exhibited size reduction (miniaturization) while maintaining their characteristic eccentricity. These adapted cells sustained a negative phototactic response among the remaining motile population, supporting optical controllability despite reduced swimming speed. These results indicate that stepwise acclimation allows *E. gracilis* to retain partial motility and phototactic controllability under simulated physiological saline conditions, and that the observed miniaturization and preserved photo-responsiveness may be useful features for future biohybrid microrobotics.

## 1. Introduction

Minimally invasive surgery, localized drug delivery systems (DDSs), and diagnostic procedures require microrobots capable of navigating inside the human body [1,2,3,4]. Both natural and artificial microrobots exist; however, artificial types face technical challenges in power sources and autonomous control in response to external stimuli. In contrast, bio-hybrid types utilizing living cells offer advantages such as environmental nutrient harvesting, self-replication, autonomous control, and efficient propulsion through biological energy conversion [5,6]. Among various methods—including magnetic, optical, and acoustic fields—light-driven control is particularly advantageous due to its high spatial selectivity and controllability [7,8,9,10]. Several microorganisms, such as *Artemia* [11,12], *Volvox* [13,14], and *Chlamydomonas* [15,16], exhibit dynamic responses to light stimulation, but size constraints limit viable options. *Artemia* (body length > 1 mm) and *Volvox* (200–500 µm) are too large for intravascular navigation. Chlamydomonas and *Euglena gracilis* (*E. gracilis*) present appropriate dimensions. *Chlamydomonas* presents drawbacks: unstable phototactic direction—where positive phototaxis occurs under conditions expected for negative response—and mandatory chemical pretreatment [17,18]. *E. gracilis* (approx. 50 µm length, 10 µm width) is a suitable candidate for in vivo applications due to its high biocompatibility [19,20]. Biocompatible micro-platforms derived from sperm or pollen require chemical modification and operate via magnetic drive. Micro-platforms derived from bacteria also exist, which require genetic engineering from the perspective of toxicity. Compared to these, *E. gracilis* exhibits immediate negative phototaxis under strong light without chemical modification [21,22,23].

Previous studies have reported systems that dynamically control the shape of *E. gracilis* populations using patterned light to transport micro-objects [24]. Additionally, as demonstrations of complex biomedical tasks, light-controlled *E. gracilis*-based bio-microrobots capable of crossing the intestinal mucosa [25], as well as precise control of moving direction and morphological changes via photonic nanojets [26], have been reported. However, applying microorganisms in vivo presents obstacles, primarily the need to prevent elimination by the immune system and to verify operation within the physiological environment. To prevent immune clearance, coating algae or *E. coli* with biomimetic materials has been proposed [27,28]. Yet, the fundamental verification of the function of *E. gracilis* in fluids that simulate the in vivo environment remains insufficient. Cultures using physiological saline or sodium chloride have been conducted. These studies have been limited to use as control groups for medium comparison or for structural analysis under salt stress; few have focused on their motility function as robots.

Phosphate-Buffered Saline (PBS) served as the biological surrogate fluid due to its high pH buffering capacity and osmolarity (~280–300 mOsm/L), making it suitable for stably mimicking the in vivo environment, such as interstitial fluid. Richter et al. reported significant impairment of *E. gracilis* swimming speed when NaCl concentration exceeds 10 g/L [29]. Since the total salt concentration of the PBS solution used in the present study is approximately 9.9 g/L, direct transfer to this environment presents a risk of significantly degrading motor function. However, given that *E. gracilis* possesses the ability to adapt to environmental changes [30], we hypothesize that by undergoing a stepwise acclimatization process to increasing salt concentrations, *E. gracilis* can maintain its motility and phototactic controllability as a microrobot in high-salt environments.

To evaluate the effects of both direct transfer and stepwise acclimatization, this study employed mixtures of Hutner medium (HUT)—a standard culture solution—with three types of solutions: PBS, NaCl, and FBS. For each combination, five types of media with stepwise varied mixing ratios enabled a comparative analysis under conditions close to the physiological environment. The evaluation focused on the major axis length and eccentricity of the cells, in addition to swimming speed and negative phototactic response rate, which are essential for optical driving. Since the hydrodynamic propulsion resistance of an ellipsoid moving through water depends not only on swimming speed but also on its major axis and eccentricity (where resistance increases as the major axis lengthens and decreases as eccentricity approaches 1) [31], this analysis included measurements of these morphological parameters.

## 2. Materials and Methods

### 2.1. E. gracilis Strain and Culture Conditions

Hutner medium (HUT) maintained the culture of *E. gracilis* strain NIES-48 (National Institute for Environmental Studies, Tsukuba, Japan), with subculturing performed every four weeks. An incubator (ITBOX-S-4B+, ITPlants Co., Ltd., Otsu, Shiga, Japan) maintained conditions at 24 °C and 25% humidity under a 12 h light/dark cycle (1000 lx).

**Figure 1:**
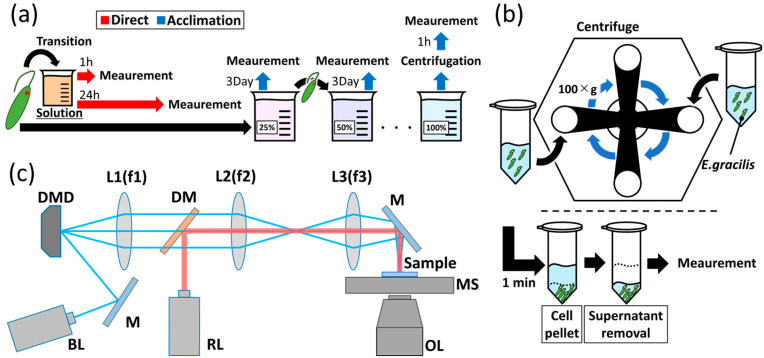
Experimental setup and procedure. (**a**) Timelines for direct transfer and stepwise acclimation experiments (mixing ratios of PBS, NaCl, or FBS solutions in HUT: 0, 25, 50, 75, and 100%). (**b**) Sample preparation via centrifugation. (**c**) Optical system for phototaxis measurement. BL: Blue laser (stimulation light); RL: Red laser (background light); DMD: Digital Micromirror Device; L1–L3: Lenses; M: Mirror; DM: Dichroic mirror; MS: Microscope stage; OL: Objective lens.

We prepared five test media for each solution with mixing ratios of HUT and PBS (PBS 7.4, Gibco, Thermo Fisher Scientific, Waltham, MA, USA. Components: sodium chloride, sodium phosphate dibasic, potassium phosphate monobasic), NaCl solution (137 mM, equivalent to the NaCl concentration in PBS), or FBS (SERANA (Serana Europe GmbH), SRN S-FBS-NL-015, Pessin, Germany) at 0, 25, 50, 75, and 100% (*v*/*v*) and conducted evaluations using the following two methods: The first method involved directly transferring cells into test solutions at each concentration. Direct immersion of cells in each test solution allowed parameter measurements at 1 and 24 h. As the second method, stepwise acclimatization adapted cells to the environment of each solution. The concentration of each solution increased in increments of 25% (0% → 25% → 50% → 75% → 100%). At each stage, a 3-day static culture under the aforementioned conditions preceded the transfer to the next concentration (Figure 1a), followed by measurements 1 and 24 h after transfer to the final target solution (100% concentration of each solution). Subsequent analysis examined the motility and phototaxis of both the direct transfer and acclimatization groups using the methods described later. Before measurements, centrifugation (H-19α, KOKUSAN, Saitama, Japan) at 100× *g* for 1 min concentrated the samples (Figure 1b), which were then loaded into a hemocytometer (177-212C, Watson Co., Ltd., Tokyo, Japan) after supernatant removal.

We defined the PBS ratio x (0 ≤ x ≤ 1.0) as the independent variable, which is proportional to the osmotic pressure P experienced by the cells. HUT contains trace minerals including KH_2_PO_4_, MgSO_4_, and minor salts; see Ref. [32] for the full composition. The contribution of these trace salts is negligible compared to that of PBS. Furthermore, HUT medium has negligible ionic strength compared to PBS. Therefore, the PBS ratio x is the dominant factor determining the osmotic pressure.

### 2.2. Optical System

The detailed configuration and specifications of this system are described in a previous report [24] (Figure 1c). A digital micromirror device (DMD) reflected the stimulation light from a blue laser (453 nm) as arbitrary light patterns. A relay lens system and a dichroic mirror projected these patterns onto the sample plane. For background illumination, a red LED (625 nm) provided light to avoid inducing a phototactic response in *E. gracilis* [33,34]. An objective lens (MRH00045 ×4, Nikon, Tokyo, Japan) facilitated the observations.

### 2.3. Cell Morphology Analysis

Image and video data acquired with a digital microscope (VHX-7000, KEYENCE, Osaka, Japan) were analyzed using a custom Python program executed in Google Colab. The analyses were performed in Python 3.12.12 or 3.12.13, depending on the Colab runtime environment, and OpenCV 4.13.0 was used for image processing. The algorithm quantified cell morphology and motility parameters as follows. For morphological analysis, individual cells were extracted from still images using the following procedure:

1. Image preprocessing and cell region extraction

A Gaussian filter (5 × 5 kernel) reduced image noise, and RGB-to-HSV conversion served to exploit the characteristic chlorophyll-derived hue of *E. gracilis* (H: 25–95°). Additionally, CLAHE was applied to the V channel to normalize field illumination heterogeneity prior to thresholding. Thresholding based on *E. gracilis* pigment characteristics (H: 25–95°) segmented the cells, and morphological operations on the binary images removed minute noise to extract cell contours.

2. Calculation of shape parameters

**Figure 2:**
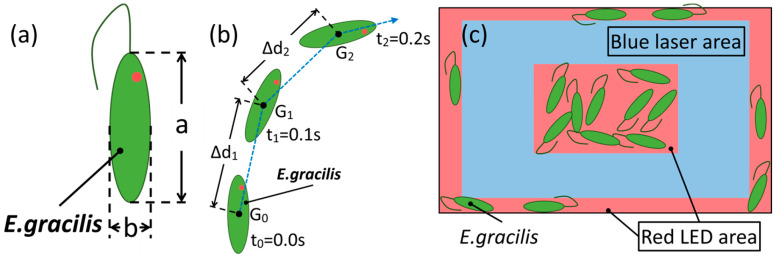

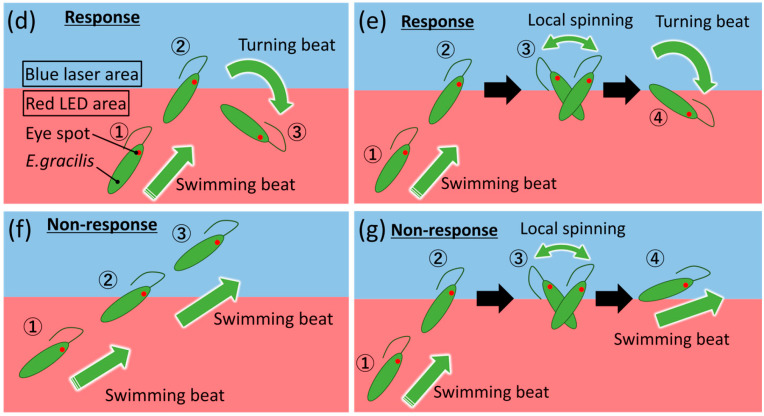
Schematic of the image analysis algorithm and phototaxis evaluation system. (**a**) Definition of morphological characteristics (*a*: major axis; *b*: minor axis). (**b**) Calculation model of swimming speed. Instantaneous speed is calculated based on the travel distance (Δ*d*) of the *E. gracilis* centroid and the elapsed time (Δ*t* = 0.1 s) between consecutive frames. (**c**) Light irradiation pattern for the negative phototaxis test. A blue laser (453 nm) projects a hollow rectangular pattern surrounding a central non-irradiated region (0.7 × 0.35 mm) to form an optical boundary. (**d**–**g**) Definition of behavioral classification in the boundary region. (**d**) Response (avoidance). (**e**) Response with local spinning. (**f**) Non-response (entry). (**g**) Non-response with local spinning.

Ellipse fitting to the extracted cell contours determined the major axis *a* and minor axis *b*. The following equation calculates eccentricity *e*, an indicator of cell shape (Figure 2a):

(1) $$e=\sqrt{1-{\left(\frac{b}{a}\right)}^{2}}$$

3. Outlier removal via multi-stage filtering

A three-stage screening process removed inappropriate targets. Filtering based on extreme dimensions (area, major axis) eliminated image processing noise and debris. The bottom 10th percentile (*e* < 0.678) was excluded as a shape-based filter, as cells with near-spherical morphology degrade ellipse-fitting accuracy and do not represent the characteristic spindle form of *E. gracilis*. Statistical outlier removal applied distinct criteria for size and shape. Log-transformation of the major and minor axes (assuming a log-normal distribution) excluded individuals outside the mean ± 3σ range. For eccentricity (assumed normal), the filter removed individuals outside the mean ± 2σ range.

4. Statistical analysis

Three independent trials provided the data for each condition (n = 3). Digital microscopy (×50 objective) captured one field to analyze all filtered individuals, and the mean values from each trial served as independent data points. To measure swimming speed, a three-stage algorithm tracked individual cells for motility analysis using 12 s video data (objective lens magnification: ×100):

### 2.4. Swimming Speed and Motility Rate

1. Size filtering using the Log-Otsu method

A preliminary scan of the target video automatically determined the optimal size threshold for detection targets. Otsu’s method applied after logarithmic transformation—necessitated by the wide distribution of detected object areas—defined the boundary separating minute noise from cell clusters. Calculation of the mean μ_log_ and standard deviation σ_log_ for the logarithmic area distribution established the normal size criterion as μ_log_ ± 3σ_log_. The process tracked only individuals meeting this criterion.

2. Individual tracking and trajectory smoothing

For cell extraction in each frame, image moments calculated centroids, supplementing the preprocessing steps (CLAHE, HSV binarization) used in static image analysis. A nearest neighbor search algorithm tracked multiple individuals by linking centroids with the shortest distance within a search radius of *r* < 60 px between consecutive frames (Δ*t* = 0.1 s). A moving average filter with a window size of *w* = 5 was applied to the coordinate data (*x*, *y*) to smooth the trajectories, removing minute vibration noise caused by Brownian motion or digital image quantization errors.

3. Calculation of mean speed and motility activity

Calculation of movement distance *Δd* from smoothed trajectory data determined the instantaneous speed *v*_inst_ and the mean swimming speed of each individual as the average over the entire tracking period, including stop periods (Figure 2b). We defined the motility rate *R*_m_ (%) to quantitatively evaluate the activity of the entire population. Previous studies have reported that the swimming speed of *E. gracilis* is 50 µm/s or higher [35,36]. A threshold of *v*th = 5.0 µm/s—corresponding to 1/10 of the reported value—classified individuals exceeding this value as “Active.” *R*_m_ was calculated as the ratio of active frames *N*_active_ to total observed frames *N*_total_:

(2) $$R_{m}=\frac{\Sigma_{i=1}^{M}\;N_{active,i}}{\Sigma_{i=1}^{M}\;N_{total,i}}\times 100$$

where *i* denotes each tracked individual, and *M* is the total number of tracked individuals. A statistical filter (mean ± 3σ) was applied to the velocity distribution of all individuals, excluding data outside this range to calculate the final mean speed of the population.

4. Statistical analysis

Three independent trials provided the data for each condition (n = 3). The analysis included all trackable individuals captured in the video per trial, regardless of swimming status, and the mean values obtained from each trial served as independent data points.

### 2.5. Phototaxis Assay

To quantitatively evaluate negative phototaxis, the optical system described above projected a hollow rectangular pattern of blue laser light (453 nm) onto the *E. gracilis* suspension on the hemocytometer (Figure 2c) (outer dimensions: approx. 1.4 mm × 0.7 mm; central non-irradiated region: approx. 0.7 mm × 0.35 mm). To calculate the response rate, video analysis tracked the behavior of individuals swimming in the inner non-irradiated region when they reached the boundary with the blue laser irradiated region. Cells that exhibited avoidance behavior upon reaching the boundary were defined as ‘Response’ (Figure 2d), whereas those that entered the region directly were defined as ‘Non-response’ (Figure 2f). According to a previous report [22], switching to flagellar movement characteristic of negative phototaxis occurs 0.23 ± 0.06 s after receiving strong light stimulation. *E. gracilis* exhibits a behavior called localized spinning (rotation on the spot) in response to strong light stimulation. Although this represents a physiological response to light, region entry solely determined the classification, regardless of the presence of spinning behavior (Figure 2e,g).

The following equation determines the negative phototactic response rate *R*_neg_ as the ratio of the number of avoidances *C*_avoid_ to the total number of observed contacts *C*_total_ based on manual measurements:

(3) $$R_{neg}=\frac{C_{avoid}}{C_{total}}$$

This analysis examined the behavior of 30 randomly selected individuals per trial across three trials (n = 3), treating the value obtained from each trial as a single independent data point to calculate the mean and standard deviation.

## 3. Results

### 3.1. Direct Transfer: Morphology and Motility

**Figure 3:**
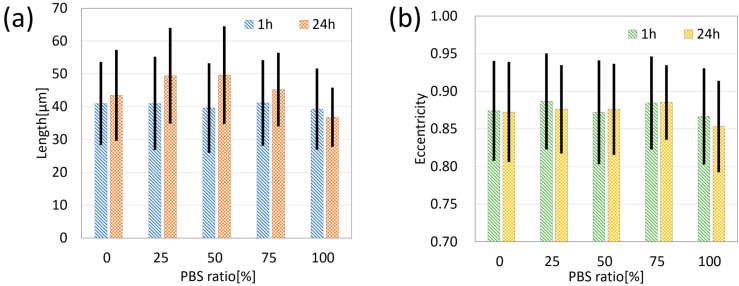
Morphological characteristics of *E. gracilis* in the direct transfer group. Major axis (**a**) and eccentricity (**b**) of *E. gracilis* 1 and 24 h after direct transfer to HUT/PBS mixtures (0, 25, 50, 75, and 100%). Data represent mean ± SD (n = 3).

As shown in Figure 3, direct transfer to high-concentration PBS induced a biphasic morphological response—initial swelling followed by shrinkage—while preserving the fundamental cell shape. Among the morphological characteristics, the major axis a showed no concentration-dependent changes after 1 h and exhibited an upward-convex trend peaking at PBS 25% and 50% after 24 h (Figure 3a). Values under all conditions at 1 h ranged from approximately 39–41 µm, comparable to the control group (PBS 0%: 40.9 µm). The major axis a at 24 h exhibited a non-monotonic trend, peaking at intermediate PBS concentrations (25–50%) before declining at higher concentrations (≥75%). This convex pattern suggests a biphasic response: initial swelling due to osmotic adjustment, followed by shrinkage under severe osmotic stress. Eccentricity e remained unaffected by PBS concentration or immersion time at both 1 h and 24 h (Figure 3b). The control group (PBS 0%) displayed an eccentricity of *e* = 0.87, and values under all conditions remained within the range of *e* = 0.85–0.89.

**Figure 4:**
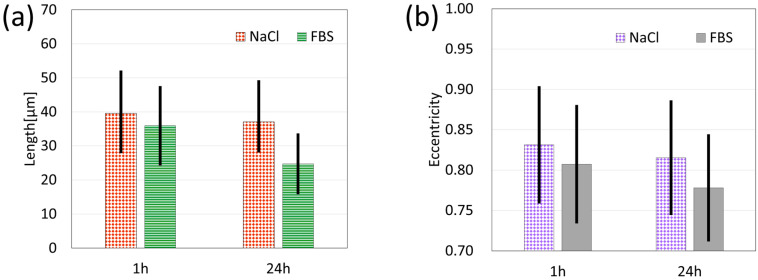
Morphological characteristics of *E. gracilis* in the direct transfer group to NaCl and FBS. (**a**) Major axis length and (**b**) eccentricity of *E. gracilis* 1 and 24 h after direct transfer. Data represent mean ± SD (n = 3).

The NaCl and 100% FBS solutions revealed differences in the behavior of the major axis *a* (Figure 4). In the NaCl solution, the major axis *a* showed only a slight decrease from 39.6 µm at 1 h to 37.0 µm at 24 h, whereas in the 100% FBS solution, it decreased from 35.9 µm to 24.7 µm. The eccentricity *e* remained within the range of approximately 0.78–0.83 in both solutions, showing no significant variation.

**Figure 5:**
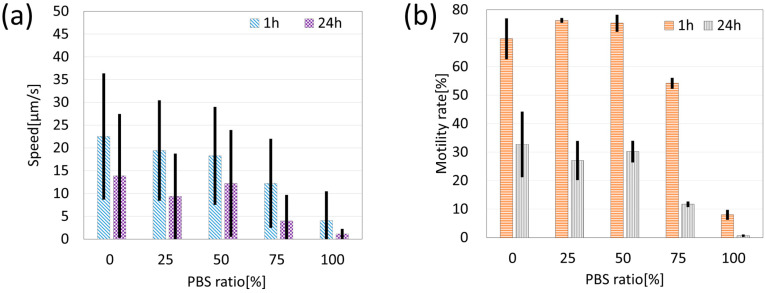
Motility characteristics of *E. gracilis* in the direct transfer group. (**a**) Swimming speed and (**b**) motility rate. Measurements of both parameters 1 and 24 h after direct transfer to HUT/PBS mixtures (0, 25, 50, 75, and 100%). Data represent mean ± SD (n = 3).

Videos S1 and S2 show representative original and analyzed videos for swimming speed measurements. Swimming speed and motility rate showed a clear decreasing trend with increasing PBS concentration at both time points (Figure 5), indicating swimming inhibition induced by salt stress. At 1 h, the mean speed declined from 22.5 ± 13.8 µm/s at PBS 0% to 4.1 ± 6.4 µm/s at PBS 100%. Linear regression yielded *v*(x) = −17.6x + 24.1 (R^2^ = 0.858, *p* < 0.001), indicating a strong negative correlation between PBS concentration and swimming speed. The slope (−17.6 µm/s per unit PBS ratio) indicates that each 25% increase in PBS concentration reduces the swimming speed by approximately 4.4 µm/s. At 24 h, a similar trend was observed (*v*(*x*) = −12.3*x* + 14.3, R^2^ = 0.700, *p* < 0.001), though the rate of decline was slightly reduced compared to at 1 h. The mean individual speed fell below 5.0 µm/s under the conditions of PBS 75% (24 h) and PBS 100% (1 h and 24 h). The motility rate remained low at less than 10% under high-concentration (PBS 100%) conditions after both 1 h and 24 h (Figure 5b). Increasing PBS concentration correlated with a decreasing trend, and the rate reached 0.8% after 24 h, indicating that almost all individuals had ceased swimming. Elapsed time led to lower motility rates, remaining below 40% under all conditions at the 24 h mark.

**Figure 6:**
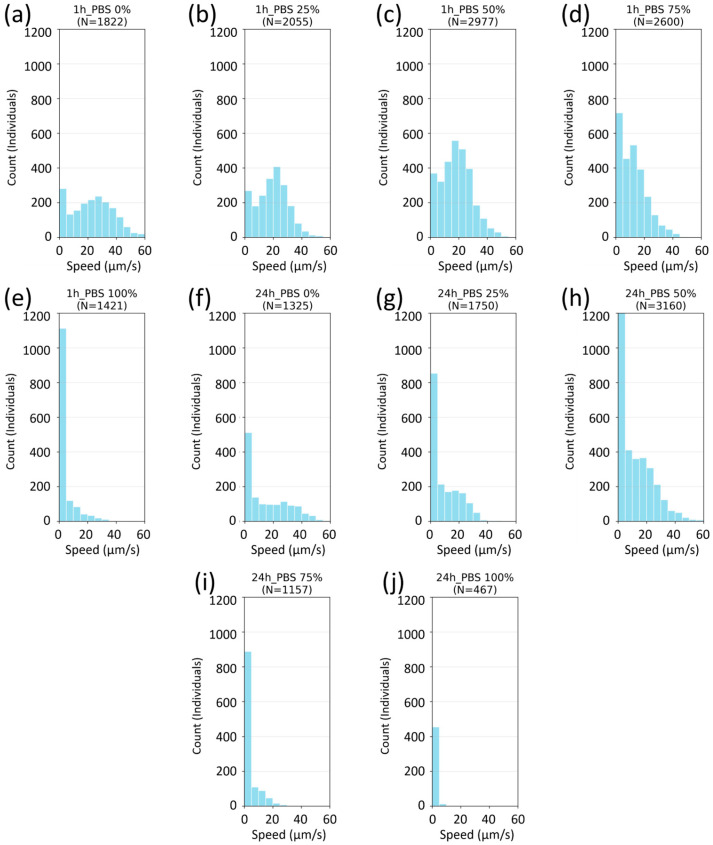
Swimming speed distribution and motility rate of the direct transfer group. Distributions and motility rates in HUT/PBS mixtures (0, 25, 50, 75, and 100%) at 1 h and 24 h after transfer (PBS concentrations: 0, 25, 50, 75, and 100%): (**a**) 1 h, PBS 0%; (**b**) 1 h, PBS 25%; (**c**) 1 h, PBS 50%; (**d**) 1 h, PBS 75%; (**e**) 1 h, PBS 100%; (**f**) 24 h, PBS 0%; (**g**) 24 h, PBS 25%; (**h**) 24 h, PBS 50%; (**i**) 24 h, PBS 75%; (**j**) 24 h, PBS 100%.

The swimming speed distribution at 1 h was bimodal (Figure 6), indicating that high osmotic pressure acts as a selective filter that segregates the population into a small fraction of salt-tolerant swimmers and a majority of immobilized individuals. The distribution showed a peak at approximately 20–30 µm/s with a relatively narrow spread. At higher PBS concentrations (≥75%), the distribution not only shifted leftward but also showed a marked increase in the frequency of near-zero velocities (*v* < 5 µm/s), which reflects a growing fraction of immobilized cells. At 24 h, the near-zero velocity fraction dominated across all concentrations, a pattern consistent with weakening of the cells due to prolonged salt stress. At PBS 100%, almost no individuals with a swimming speed exceeding 10 µm/s were observed.

**Figure 7:**
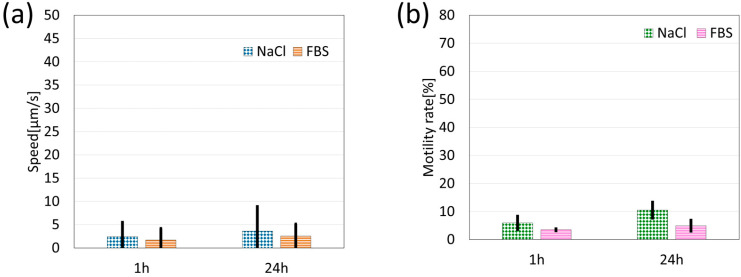
Motility characteristics of *E. gracilis* in the direct transfer group to NaCl and FBS. (**a**) Swimming speed and (**b**) motility rate of *E. gracilis* 1 and 24 h after direct transfer. Data represent mean ± SD (n = 3).

In the NaCl and 100% FBS solutions, swimming speeds remained below 5.0 µm/s across all measurements at both 1 h and 24 h (Figure 7). For the motility rate, although a recovery trend was observed in both solutions at 24 h, it remained low at 10.5% in the NaCl solution and 5.0% in the 100% FBS solution.

### 3.2. Direct Transfer: Phototaxis

**Figure 8:**
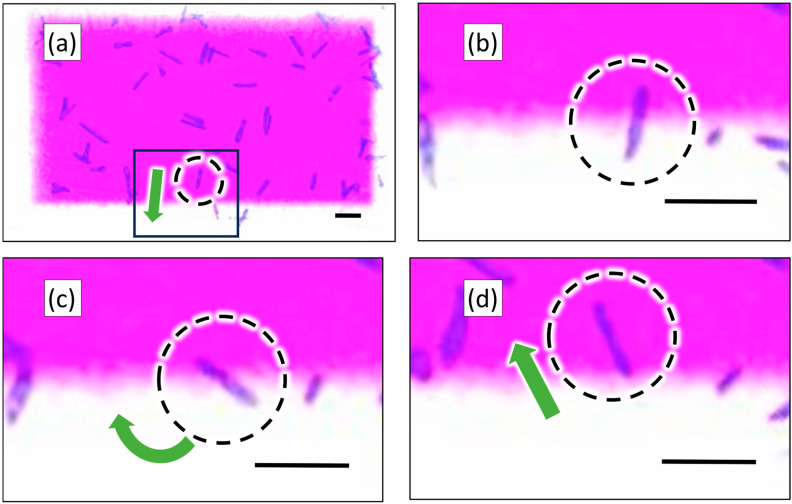
Negative phototactic response of *E. gracilis*. Representative sequential images from an experimental video (Scale bar = 25 μm). (**a**) Immediately before entering the region irradiated with high-intensity laser light. (**b**) Entering the high-intensity light region. (**c**) Manifestation of negative phototactic response (change of direction). (**d**) Escape behavior from the high-intensity light region.

**Figure 9:**
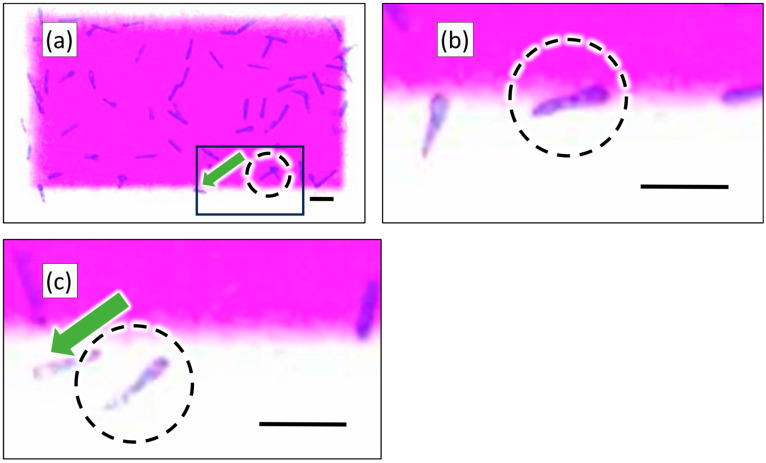
Non-response behavior of *E. gracilis*. Representative sequential images from an experimental video (Scale bar = 25 μm). (**a**) Immediately before entering the high-intensity laser light region. (**b**) Entering the high-intensity light region. (**c**) Swimming straight through the high-intensity light region without showing negative phototaxis.

**Figure 10:**
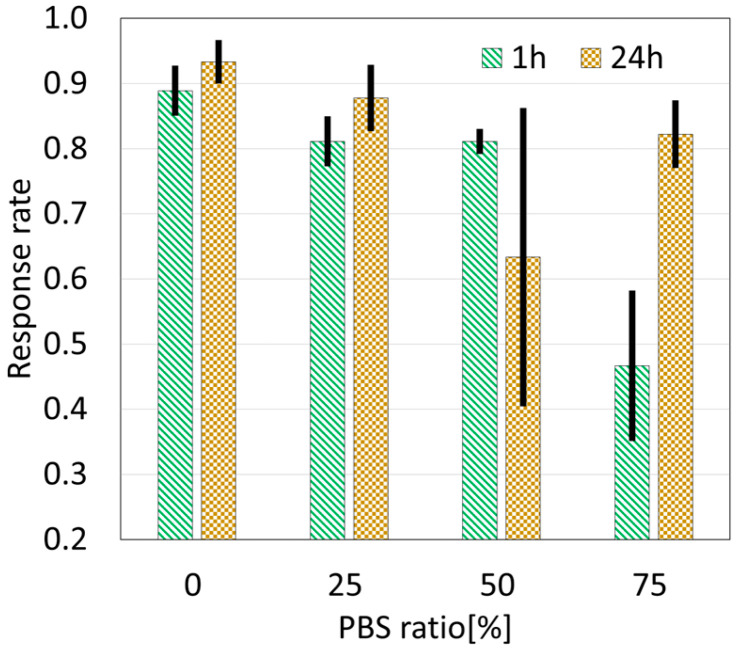
Negative phototactic response rate of *E. gracilis* in the direct transfer group. Proportion of individuals showing negative phototaxis in HUT/PBS mixtures (0, 25, 50, and 75%) measured at 1 and 24 h. Data represent mean ± standard deviation (SD). Observation of 30 individuals per independent measurement (n = 3).

Figure 8 (Response) and Figure 9 (Non-response) present excerpts from the phototaxis assay video (Video S3). Individuals that entered the irradiated region and turned back to the non-irradiated region before their entire body completely entered were counted as a ‘response’, whereas those that did not were counted as a ‘non-response’. As shown in Figure 10, at both 1 h and 24 h, the response rate remained stable at low PBS concentrations, whereas large fluctuations were observed at high PBS concentrations. These fluctuations indicate that elevated PBS concentrations may suppress the negative phototactic response. The low concentration range (PBS 25%: *R*_neg_ = 0.81 (1 h), 0.88 (24 h)) maintained high response rates, reaching approximately 90% of the control group levels (PBS 0%: *R*_neg_ = 0.89 (1 h), 0.93 (24 h)) (Figure 10). Medium and high concentration ranges exhibited a general decreasing trend with increasing concentration, showing marked decreases such as 0.53 at PBS 50% (24 h) and 0.47 at PBS 75% (1 h). Linear regression analysis of the data up to 75% PBS concentration yielded *R*_neg_ = −0.507*x* + 1.06 (R^2^ = 0.680, *p* < 0.001), indicating a significant negative correlation. The slope (−0.507 per unit PBS ratio) suggests that each 25% increase in PBS concentration results in approximately a 0.13 reduction in the response rate. Large fluctuations, such as a recovery from PBS 50% to PBS 75%, resulted in no significant difference after 24 h (*p* = 0.138). The extremely low number of swimming individuals in the PBS 100% solution precluded quantitative evaluation of phototaxis (Figure 5b).

As with the 100% PBS solution, the 100% FBS solution contained too few swimming cells to permit quantitative evaluation of phototaxis. In contrast, the NaCl solution retained a sufficient number of swimming individuals and maintained a high negative phototactic response (*R*_neg_ = 0.92 at 1 h and 0.88 at 24 h after transfer), comparable to the control group.

### 3.3. Acclimation: Morphology and Motility

**Figure 11:**
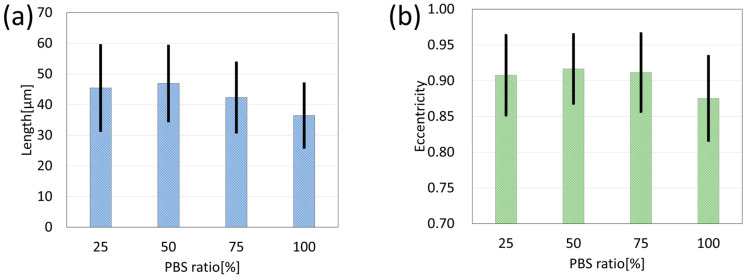
Morphological characteristics of *E. gracilis* in the acclimation group. (**a**) Major axis and (**b**) eccentricity measured after 3-day incubation at each PBS concentration (25, 50, and 75%) and 1 h after transfer to 100% PBS. Data represent mean ± SD (three independent cell lots, n = 3 measurements each).

To overcome the near-total loss of motility observed in direct transfer, we evaluated a stepwise acclimation process. As illustrated in Figure 11, this approach resulted in progressive cell miniaturization without altering the fundamental cell shape. The major axis a dropped from 45.4 µm at PBS 25% to 36.5 µm at PBS 100%. Linear regression yielded *a*(*x*) = −12.60*x* + 50.65 (R^2^ = 0.286, *p* < 0.001), indicating a significant negative correlation between PBS concentration and the major axis. The slope corresponds to a 3.15 µm reduction in the major axis for every 25% increase in PBS concentration, consistent with shrinkage due to stepwise nutrient deficiency. Eccentricity, *e*, remained within the range of 0.87–0.92 under all conditions (Figure 11b). Measurements of *e* = 0.91 at PBS 25% and 75%, 0.92 at 50%, and 0.88 at 100% indicated no discernible pattern.

**Figure 12:**
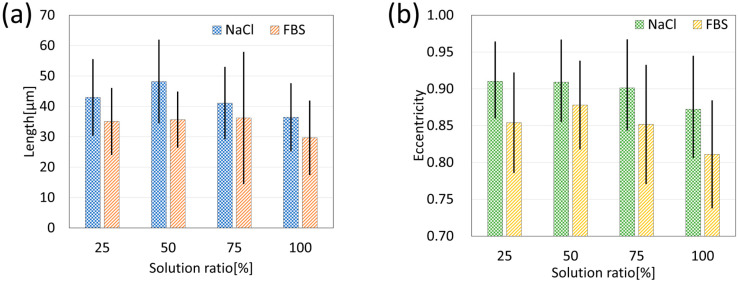
Morphological characteristics of *E. gracilis* in the acclimation transfer group to NaCl and FBS. (**a**) Major axis length and (**b**) eccentricity measured 3 days after transfer to 25, 50, and 75% mixtures, and 1 h after transfer to the 100% solution. Data represent mean ± SD (n = 3).

Figure 12 shows that stepwise acclimation reduced the major axis in both NaCl and FBS. The decrease was significant in NaCl, with a slope of 2.76 µm per 25% increase in concentration (*p* = 0.043), while FBS showed a weaker trend of 1.55 µm per 25% increase (*p* = 0.059). Eccentricity remained stable in NaCl (0.87–0.92), but decreased in 100% FBS to approximately 0.81, suggesting a more rounded morphology.

**Figure 13:**
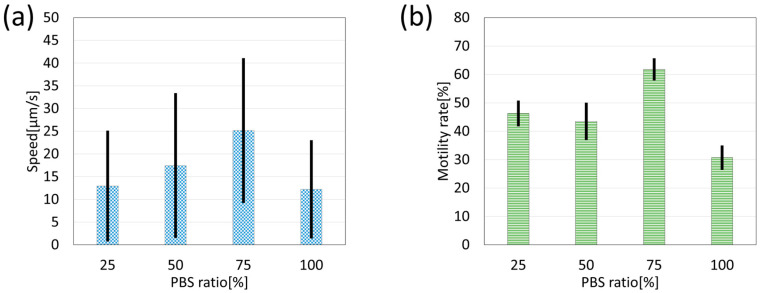
Motility characteristics of *E. gracilis* in the acclimation group. (**a**) Swimming speed and (**b**) motility rate. Measurements of both parameters after 3-day incubation at each PBS concentration (25, 50, and 75%) and 1 h after transfer to 100% PBS. Data represent mean ± SD (three independent cell lots, n = 3 measurements each).

**Figure 14:**
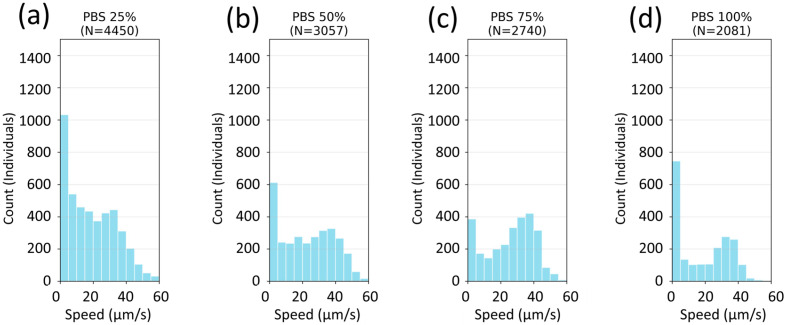
Swimming speed distribution of *E. gracilis* under stepwise PBS acclimation. Distributions after 3-day incubation at each PBS concentration (25, 50, and 75%) and 1 h after transfer to 100% PBS. (**a**) PBS 25% (Day 3); (**b**) PBS 50% (Day 6); (**c**) PBS 75% (Day 9); (**d**) PBS 100% (1 h after transfer).

Figure 13 shows that, during stepwise PBS acclimation, the mean speed increased up to 75% PBS and decreased after transfer to 100% PBS. The motility rate followed a similar pattern, although the value at 25% PBS deviated from this trend. The speed distributions in Figure 14 indicate that these changes were mainly associated with changes in the fraction of near-zero-speed cells (*v* < 5 µm/s), rather than a marked change in the swimming capacity of active cells. Specifically, the mean speed increased from 12.9 µm/s at PBS 25% to 25.2 µm/s at PBS 75%, before decreasing to 12.2 µm/s at PBS 100%. Although the motility rate was lowest at PBS 100% (30.7%), it remained higher than that in the direct transfer group.

**Figure 15:**
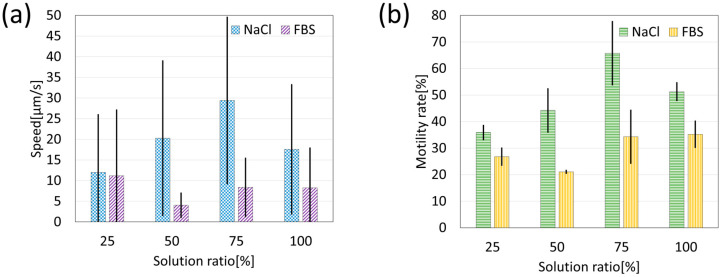
Motility characteristics of *E. gracilis* in the acclimation transfer group to NaCl and FBS. (**a**) Swimming speed and (**b**) motility rate. Measurements of both parameters 3 days after transfer to 25, 50, and 75% mixtures, and 1 h after transfer to the 100% solution. Data represent mean ± SD (n = 3).

In both NaCl and FBS, the mean speed and motility rate showed similar trends within each solution (Figure 15). The parallel behavior of these two parameters implies that the average speed was largely influenced by the fraction of motile cells, rather than by large changes in the swimming capacity of individual active cells. In NaCl, both the mean speed and motility rate increased up to the 75% mixture and then decreased, reaching 17.6 µm/s and 51.3%, respectively, in the 100% solution. The control group, which was subcultured from the same population and measured at the same time, showed a mean speed of 20.6 µm/s and a motility rate of 44.9%, yielding comparable results. This trend was also similar to that observed in PBS (Figure 13), suggesting that the motility changes in PBS were mainly attributable to its NaCl component.

In FBS, the mean speed in the 100% solution was 8.2 µm/s. Although it decreased markedly in the 50% solution, it remained around 10 µm/s across the other concentrations. The motility rate gradually increased and reached its maximum of 35.2% in the 100% solution. Compared with the direct transfer group, both the mean speed and motility rate were improved.

Measurements were also performed 24 h after transfer to the 100% solutions. In NaCl, the mean speed and motility rate showed no clear difference from the values at 1 h, whereas in FBS, they increased to 20.6 µm/s and 45.2%, respectively. However, the number of measured individuals remained low in both solutions, and the total count for each condition sometimes did not exceed 100 even when the three trials were combined.

### 3.4. Acclimation: Phototaxis

**Figure 16:**
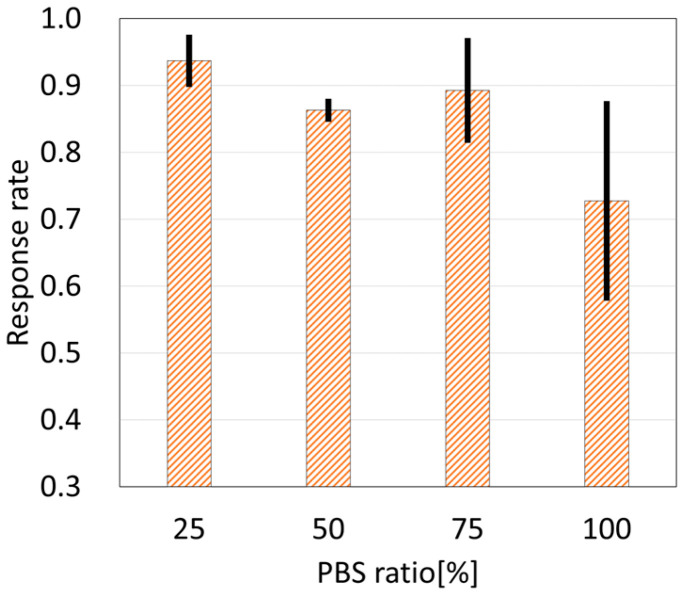
Negative phototactic response rate of *E. gracilis* under stepwise PBS acclimation. Proportion of individuals showing negative phototaxis after 3-day incubation at each PBS concentration (25, 50, and 75%) and after transfer to 100% PBS. Data represent mean ± SD. Observation of 30 individuals per independent measurement (three independent cell lots, n = 3 measurements each).

During stepwise PBS acclimation, in contrast to swimming speed, the negative phototactic response rate showed a significant decrease with increasing concentration (*p* ≈ 0.001, *R*^2^ ≈ 0.303) (Figure 16). This decoupling between motility (speed) and controllability (phototaxis) suggests that osmotic stress appears to affect motor output more strongly than the sensory pathway underlying the directional photoresponse. The light-activated adenylyl cyclase pathway [33] mediating photoresponse may exhibit a different osmotic sensitivity than the flagellar motor machinery. The response rate at mixing ratios between 25% and 75% was *R_neg_* = 0.86–0.94, whereas in the 100% solution, the response rate remained at only 80% of those values.

**Figure 17:**
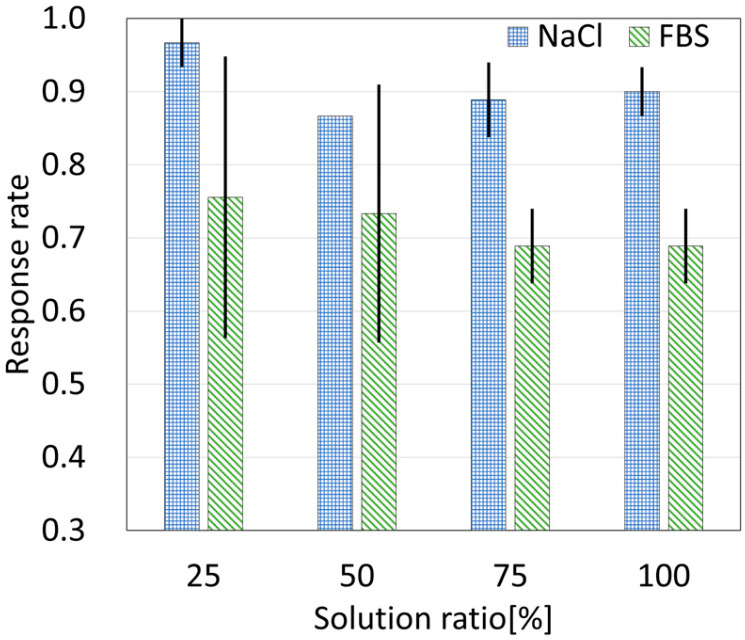
Negative phototactic response rate of *E. gracilis* under stepwise NaCl and FBS acclimation. Proportion of individuals showing negative phototaxis after 3-day incubation at each concentration (25, 50, and 75%) and after transfer to the 100% solution. Data represent mean ± SD. Observation of 30 individuals per independent measurement (n = 3).

In both NaCl and FBS, although there was a slight decreasing trend with increasing concentration, the values remained nearly constant (Figure 17). In NaCl, *R_neg_* fluctuated around 0.9, and compared with PBS, a similar trend was observed except for the 100% solution. In FBS, *R_neg_* fluctuated around 0.7, resulting in low values regardless of the concentration.

## 4. Discussion

This study evaluated the influence of the acclimation process to simulated in vivo environments (PBS, NaCl, and FBS) on the function of *E. gracilis* as a microrobot. The major axis exhibited different trends between the two groups; the direct transfer group (1 h) showed no concentration-dependent changes, whereas the acclimation group exhibited a significant reduction in cell size with increasing PBS concentration. This growth suppression likely results from relative nutrient deficiency due to long-term exposure to the PBS environment. Eccentricity remained stable under all conditions, and the elliptical shape characteristic of *E. gracilis* persisted in the PBS environment. The constancy of eccentricity despite major axis reduction implies isotropic miniaturization—both axes shrink proportionally, preserving the spindle shape and its hydrodynamic drag characteristics. This shape-preserving reduction may be advantageous for consistent navigation in capillary-scale vessels. Both NaCl and FBS solutions showed behaviors similar to those in PBS. However, in FBS, both the major axis and eccentricity were at lower levels; this decrease in eccentricity indicates that it cannot be considered pure miniaturization.

In the acclimation group, the swimming speed showed a decreasing trend only upon transfer to the 100% solutions. This result in NaCl and PBS is consistent with the report by Richter et al. [29], which states that swimming speed decreases significantly when NaCl exceeds 10 g/L. However, the motility rate and controllability improved through the acclimation process. The motility rate in the direct transfer group dropped to 8.0% (1 h) and 0.8% (24 h) in PBS 100%, whereas the acclimation group retained 30.7%. In the NaCl and FBS solutions as well, the acclimation group showed improvements to 51.3% and 35.2%, respectively, compared to the direct transfer group. These improvements imply that stepwise acclimation may help cells tolerate progressively increasing osmotic and ionic stress, thereby preserving a higher fraction of motile cells.

The reduced motility of *E. gracilis* in 100% PBS can be attributed to combined osmotic and ionic stress, rather than to NaCl alone. High salinity disturbs cellular homeostasis through ionic and osmotic imbalance, and salt-stressed *E. gracilis* cells show growth inhibition, decreased chlorophyll content, thylakoid membrane reorganization, and paramylon accumulation [37]. Cell shape in *E. gracilis* is also closely associated with the arrangement and stability of the pellicle microtubule system [38]. Abrupt exposure to PBS may therefore affect not only osmotic and ionic homeostasis but also cell-shape stability and the mechanical execution of swimming. Phototaxis in *E. gracilis* depends on photoactivated adenylyl cyclase-related signaling, as knockdown of PAC by RNA interference suppresses both positive and negative phototaxis [39]. The reduced swimming speed and weakened phototactic response observed in PBS may thus reflect the combined effects of osmotic stress, ionic imbalance, metabolic stress, altered cell-shape regulation, and impaired flagellar motion. We did not directly measure flagellar beating, intracellular ion concentrations, metabolic state, or cell viability, so this explanation should be regarded as a plausible mechanistic interpretation rather than direct experimental evidence.

The present strategy is not expected to apply to all microalgae. It is most likely to work for species that meet the following conditions: (1) they can tolerate gradual osmotic or ionic changes, (2) they retain flagellar motility after acclimation, (3) they show a stable phototactic response, and (4) their cell body is sufficiently flexible to withstand morphological adaptation. *E. gracilis* satisfies these requirements through its wall-less, flexible body and PAC-mediated phototaxis. Therefore, other wall-less or mechanically flexible phototactic microalgae may also be candidates, although the acclimation schedule, medium composition, and light-stimulation conditions would need to be optimized for each species.

For practical application, we envision blocking the blood vessel with a thermosensitive gel [40] and introducing *E. gracilis* into the actual blood vessel where blood flow is arrested. Given that blood viscosity is approximately 4 mPa·s and flagellar swimming is unhindered up to 11.2 mPa·s [41], the swimming speeds of 12.2 and 8.2 µm/s obtained in the 100% PBS and FBS acclimation groups, respectively, are considered effective. The near-total loss of motility in the direct transfer group (0.8% at 24 h) precluded phototaxis measurement, which confirms that direct transfer renders phototactic control practically infeasible at physiological PBS concentrations. In contrast, the acclimation group maintained a negative phototactic response rate (*R*_neg_) of 0.73 in 100% PBS, corresponding to approximately 82% of the direct transfer control value (0% PBS, 1 h). Corresponding values were 0.9 and 0.7 in the 100% NaCl and 100% FBS solutions, respectively. These comparisons indicate that the stepwise acclimation process preserves the photocontrol function of *E. gracilis*. One remaining issue is the low motility rate of 30.7% in 100% PBS. Previous research [24] shows that practical use, such as transporting micro-objects, requires *E. gracilis* to retain both active motility and adequate photosensitivity, and that cells can be captured and collected by gradually reducing the diameter of a circular spot enclosed by a high-intensity blue laser. Applying this optical confinement to our acclimated cells could accumulate only the active fraction, compensating for the reduced motility rate.

## 5. Conclusions

This study investigated the effects of “direct transfer” and “stepwise acclimation” to a PBS, NaCl, and FBS mixed medium on motility and phototaxis, with the aim of applying *E. gracilis* under physiological conditions. In PBS and NaCl solutions, regardless of the increase in concentration and the acclimation process, the cell shape (eccentricity) remained stable, while the cell size (major axis) and swimming speed tended to decrease in the 100% solution. Direct transfer to each 100% solution caused a near-total loss of motor function, whereas the acclimation process improved the motility rate. The acclimation group demonstrated a stable negative phototactic response rate in high-concentration PBS and saline environments, indicating that the remaining motile cells retained phototactic controllability under these conditions.

For practical application, the decrease in swimming speed under simulated in vivo environments remains a challenge. However, the size reduction and high photo-responsiveness are useful characteristics for a micromachine operating in vivo. The observed size reduction under gradually changing osmotic and nutritional conditions also suggests a potential strategy to tune cell dimensions to match target vessel diameters prior to deployment. Future work should combine the acclimation protocol with biomimetic surface coatings to prevent immune clearance and validate locomotion and phototaxis in ex vivo vascular models.

## Data Availability

The original contributions presented in this study are included in the article/Supplementary Material. Further inquiries can be directed to the corresponding author.

## Supplementary Materials

The following supporting information can be downloaded at: https://www.mdpi.com/article/10.3390/mi17070815/s1, Video S1: Raw video of motility analysis of *E. gracilis*; Video S2: Tracked video of motility analysis of *E. gracilis*; Video S3: Phototaxis assay of *E. gracilis*.
